# Development of High-Density Genetic Maps for Barley and Wheat Using a Novel Two-Enzyme Genotyping-by-Sequencing Approach

**DOI:** 10.1371/journal.pone.0032253

**Published:** 2012-02-28

**Authors:** Jesse A. Poland, Patrick J. Brown, Mark E. Sorrells, Jean-Luc Jannink

**Affiliations:** 1 Hard Winter Wheat Genetics Research Unit, United States Department of Agriculture – Agricultural Research Service, Manhattan, Kansas, United States of America; 2 Department of Agronomy, Kansas State University, Manhattan, Kansas, United States of America; 3 Department of Crop Science, University of Illinois, Urbana, Illinois, United States of America; 4 Department of Plant Breeding and Genetics, Cornell University, Ithaca, New York, United States of America; 5 Plant, Soil and Nutrition Research Unit, United States Department of Agriculture – Agricultural Research Service, Ithaca, New York, United States of America; Nanjing Forestry University, China

## Abstract

Advancements in next-generation sequencing technology have enabled whole genome re-sequencing in many species providing unprecedented discovery and characterization of molecular polymorphisms. There are limitations, however, to next-generation sequencing approaches for species with large complex genomes such as barley and wheat. Genotyping-by-sequencing (GBS) has been developed as a tool for association studies and genomics-assisted breeding in a range of species including those with complex genomes. GBS uses restriction enzymes for targeted complexity reduction followed by multiplex sequencing to produce high-quality polymorphism data at a relatively low per sample cost. Here we present a GBS approach for species that currently lack a reference genome sequence. We developed a novel two-enzyme GBS protocol and genotyped bi-parental barley and wheat populations to develop a genetically anchored reference map of identified SNPs and tags. We were able to map over 34,000 SNPs and 240,000 tags onto the Oregon Wolfe Barley reference map, and 20,000 SNPs and 367,000 tags on the Synthetic W9784×Opata85 (SynOpDH) wheat reference map. To further evaluate GBS in wheat, we also constructed a *de novo* genetic map using only SNP markers from the GBS data. The GBS approach presented here provides a powerful method of developing high-density markers in species without a sequenced genome while providing valuable tools for anchoring and ordering physical maps and whole-genome shotgun sequence. Development of the sequenced reference genome(s) will in turn increase the utility of GBS data enabling physical mapping of genes and haplotype imputation of missing data. Finally, as a result of low per-sample costs, GBS will have broad application in genomics-assisted plant breeding programs.

## Introduction

The development of molecular markers and genomic resources in barley and wheat has always been a formidable task due the massive, complex, and, in the case of wheat, polyploid genomes [1], [2], [3]. The diploid barley genome is over 5.5 GB and the hexaploid wheat genome is roughly three times larger at 16 GB [4]. The development of new sequencing technologies has greatly increased the discovery of SNPs in many species [5], including important model and non-model crop plants such as rice [6] maize [7], soybean [8], common bean [9], and sorghum [10]. SNP discovery in the wheat D-genome predecessor, *Aegilops tauschii*, was recently completed using next-generation sequencing (NGS), marking a step forward for SNP markers in large and complex genomes [11]. The discovery of high-density molecular markers in crop species will lead to a better understanding of the genetic architecture of complex traits and its application in breeding programs for crop improvement through whole genome association studies [12], [13] and genomic selection [14], [15].

The use of genome complexity reduction combined with multiplex sequencing was first demonstrated through restriction-site associated DNA (RAD) tagging [16], [17] and NGS of the RAD tags to genetically map mutations [18]. Genotyping-by-sequencing (GBS) was developed as a simple but robust approach for complexity reduction in large complex genomes [19]. Both RAD sequencing and GBS target the genomic sequence flanking restriction enzyme sites to produce a reduced representation of the genome. The GBS library development is greatly simplified compared to that of RAD. GBS requires less DNA, avoids random shearing and size selection, and is completed in only two steps on plates followed by PCR amplification of the pooled library. The original GBS approach used a single restriction enzyme to capture the genomic sequence between restriction sites [19]. Here we extend the GBS protocol to a two-enzyme system that includes one “rare-cutter” and one “common-cutter”. When combined with Y-adapters for the common restriction site, the use of two enzymes differs from the original GBS protocol in that amplified fragments in the two-enzyme libraries will all consist of the barcoded forward adapter and the common reverse adapter. This type of library construction greatly simplifies quantification of the library prior to sequencing. The two-enzyme approach can generate a suitable and uniform complexity reduction. A form of this complexity reduction approach has been successfully applied in sequencing pools of BAC libraries for construction of physical maps [20].

RAD genotyping was recently applied in barley to identify 530 SNP markers, construct a genetic linkage map and map quantitative trait loci [21]. The original GBS approach was also applied in barley to effectively map sequence tags as dominant markers on a reference map [19]. Here we apply a two-enzyme GBS approach to barley and wheat and demonstrate the robustness of GBS for genotyping in species with large, complex, and even polyploid genomes. The development of high-density (10,000 to 100,000+ markers) in species that are lacking a reference genome will facilitate the development (anchoring and ordering) of the reference genome sequence while providing tools for genomics-assisted breeding.

## Results

### Development of a two-enzyme GBS protocol and design of *PstI* barcodes

**Figure 1:**
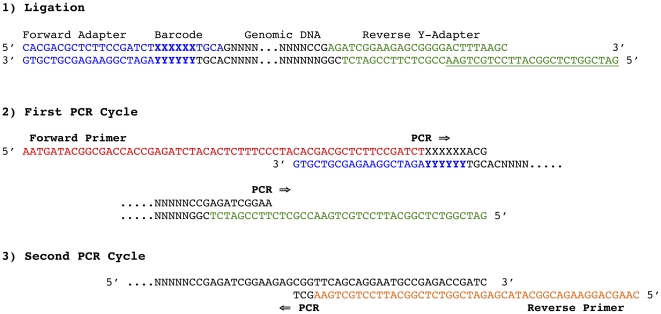
Adapter Design, PCR amplification of fragments. 1) The ligation product of a genomic DNA fragment (black) containing a *PstI* restriction site and a *MspI* restriction site. The forward adapter (blue) binds to a *PstI* generated overhang. The 4–9 bp barcode for this adapter is in bold with “X”. The *MspI* generated overhang corresponds to the reverse Y-adapter (green). The unpaired tail of the Y-adapter is underlined. 2) During the first round of PCR only the forward primer (red) can anneal. PCR synthesis of the complementary strand proceeds to the end of the fragment synthesizing the compliment of the Y-adapter tail. 3) During the second round of PCR the reverse primer (orange) can anneal to the newly synthesized compliment of the Y-adapter tail. This PCR reaction then proceeds to fill in the compliment of the forward adapter/primer on the other end of the same fragment.

We employed a two-enzyme restriction digest to generate a library consisting of DNA fragments with a forward adapter and reverse adapter on opposite ends of every fragment. The combination of a rare-cutting enzyme and a second common-cutting enzyme will produce a digest largely consisting of 1) fragments with a rare cut-site and a common cut-site or 2) fragments with two common cut-sites. A rare-cutting restriction enzyme, *PstI* (CTGCAG), was chosen based on previous success in complexity reduction in large genomes [22]. The second enzyme used here, *MspI* (CCGG), has a more common recognition site. Barcoded forward adapters were designed with the *PstI* restriction overhang while the reverse adapter matches the *MspI* overhang. To eliminate amplification of the more common *MspI-MspI* fragments, we designed the common reverse adapter as a Y-adapter. The Y-adapter contains an exact match (but no complement) to the reverse primer used in PCR amplification. During the first cycle of PCR, amplification proceeds only from primer annealing to the forward adapter. Binding sites for the reverse primer are only created during the first round of PCR by extension from forward primers on the other end of the same fragment (Figure 1). This design allows amplification of only *PstI-MspI* fragments and produces a uniform library (all fragments are Forward Adapter – genomic DNA – Reverse Adapter). (see details in Material and Methods and Text S1). PCR amplification with a short extension time (<30 s) enriches for shorter fragments suitable for bridge-amplification on the Illumina flow-cell.

To enable multiplex sequencing of the *PstI* GBS libraries, we designed a set of DNA barcodes ranging in length from 4 bp to 9 bp that balanced the base composition of the GBS library with the overhang generated by *PstI* restriction digest (Table S1). Modulation of the length of GBS barcodes while selecting barcodes with balanced sets of nucleotides at each position reduces phasing errors in the Illumina sequencing run [19]. A barcode design algorithm was written in Java to select a set of suitable barcodes for *PstI* (see Materials and Methods, Tables S1 and S2).

**Library Development and Sequencing:** To evaluate GBS in barley and wheat while developing high-density genetic maps, we utilized two bi-parental populations. For barley, the Oregon Wolfe Barley (OWB) population consists of 82 double haploid (DH) lines and is a morphologically diverse population segregating for a number of distinct traits [23], [24]. For wheat, the reference population is derived from a cross between the cultivar ‘Opata 85’ and the synthetic hexaploid W9784 (SynOpDH) and consists of 215 DH lines of which 164 were used for this study [25].

We constructed two 48-plex libraries from the OWB population and four 48-plex libraries from the SynOpDH population consisting of a single sample of each DH line and replicated samples of each parent. The libraries were sequenced on Illumina GAII or Illumina HiSeq2000. From the (unfiltered qseq) Illumina data, sequences were assigned to individual samples using the barcode sequence and trimmed to 64 bp for faster processing. Only sequences that had an exact match to a barcode followed by the expected sequence of 5 nucleotides remaining from a *PstI* cut-site were kept. Tags were defined as unique sequences within the data set and collapsed by lines.

To identify SNPs in the populations, all pairs of tags were evaluated for a one or two base-pair difference. Bi-allelic SNPs were identified by querying the filtered tags for pairs of sequences which were 1) identical except for one or two nucleotide(s), 2) present in >20% of the individuals and 3) passed a Fisher Exact test for independence. We examined independence at each possible pair to avoid paralogous SNPs which would presumably segregate independently. In contrast, allelic SNPs should be mutually exclusive in inbred lines. These tags were then designated bi-allelic SNPs with two alleles and missing data. If a SNP call was heterozygous, presumably due to sequencing errors, this call was set to missing data.

**Genetic Mapping of Tags and SNPs:** GBS SNPs were identified from sequence tags that were identical save for one or two base pairs and that passed other filtering steps. We used reference genetic maps for OWB and SynOpDH to first add biallelic GBS SNPs and then sequence tags (treated as dominant markers) to the maps. This enabled full utilization of data sets with large amounts of missing data. The reference map for OWB consists of 2,382 markers, primarily SNPs and DArT (Diversity Arrays Technology Pty Ltd) [24]. The SynOpDH reference map consisted of 1351 DArT and SSR markers [25]. We placed SNPs into recombination bins as follows. Recombination bins were defined by each observed recombination across the population. A GBS SNP was placed in a bin if the parent of origin of the SNP matched that of bin markers for all lines where the data was present. Missing data caused GBS SNPs to sometimes be placed ambiguously into more than one bin (see Materials and Methods). Tags were then mapped as dominant markers onto the bin map using a binomial test to determine the best placement [19].

**Figure 2:**
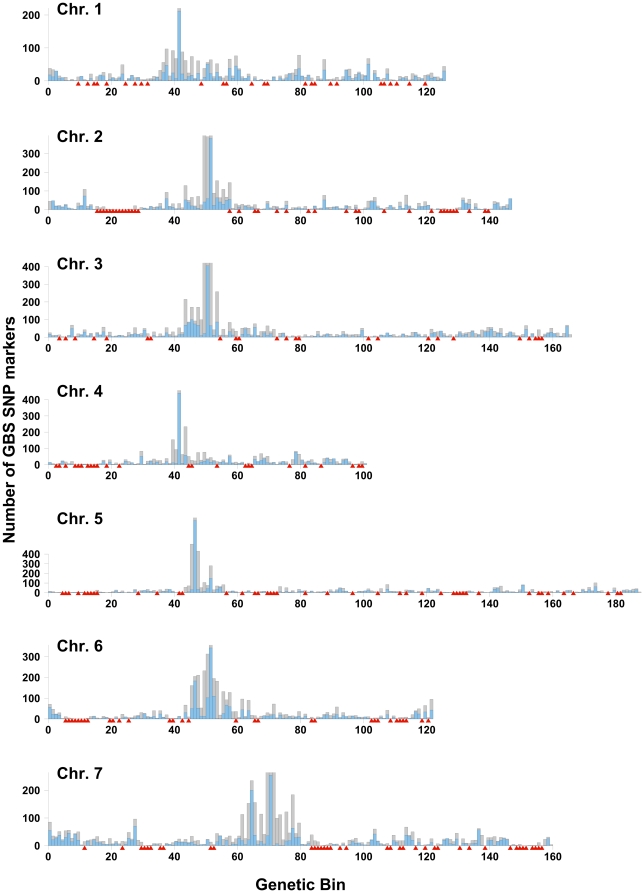
Distribution of GBS SNP markers in the Oregon Wolfe Barley (OWB) bin map. Histogram showing the number of markers from the set of GBS SNPs mapping to each bin in the OWB bin map. The number of SNPs mapping to a single bin is shown by the height of the blue bars. Additional markers that could not be placed in a single bin are show in grey. If a marker mapped to more than one bin (due to missing data), that marker was attributed to its middle bin. Bins that did not have definitive placement of any GBS SNP marker are noted with a red triangle below the plot.

Using this approach in the OWB population, we first mapped 9,545 SNPs with less than 20% missing data (Figure 2, Dataset S1). The OWB bin map with 82 DH lines contains 1019 recombination bins. From the original OWB genetic map, the parent of origin of 579 of these bins was known (i.e., one or more markers were in the bin). We were able place GBS SNPs in an additional 239 bins, increasing parent of origin resolution in the OWB population. Using this updated bin map, we then added SNPs with up to 80% missing data. Finally, the improved bin map was used to place tags from the data set as dominant markers with the binomial test. In total for the OWB population we placed 34,396 biallelic SNPs and 241,159 tags as dominant markers on the genetic map (Dataset S1).

We observed a high density of GBS markers in the likely centromeric regions of each chromosome (Figure 2). A similar observation was made on SNPs developed from expressed sequence tags [26]. We inspected the sequence of the SNP tags that were genetically mapped to the centromeric bins and did not observe repetitive sequences that would have been indicative of mapping a large number of slightly divergent centromeric (or other) repeats.

Even at the high marker density provided with GBS, we also observed several regions on the OWB map without any markers. Most notable was a region of 14 recombination bins on chromosome 2 where there was not a single informative marker. In other words, that region contains an interval for which the flanking markers recombined in 15 different lines. Given the number of lines evaluated (82), this indicates that the markers are about 18 cM apart. Yet there were no polymorphic markers in that interval enabling us to observe where recombination occurred for each line and suggesting a region of identity-by-descent (IBD) between the two parents.

**Figure 3:**
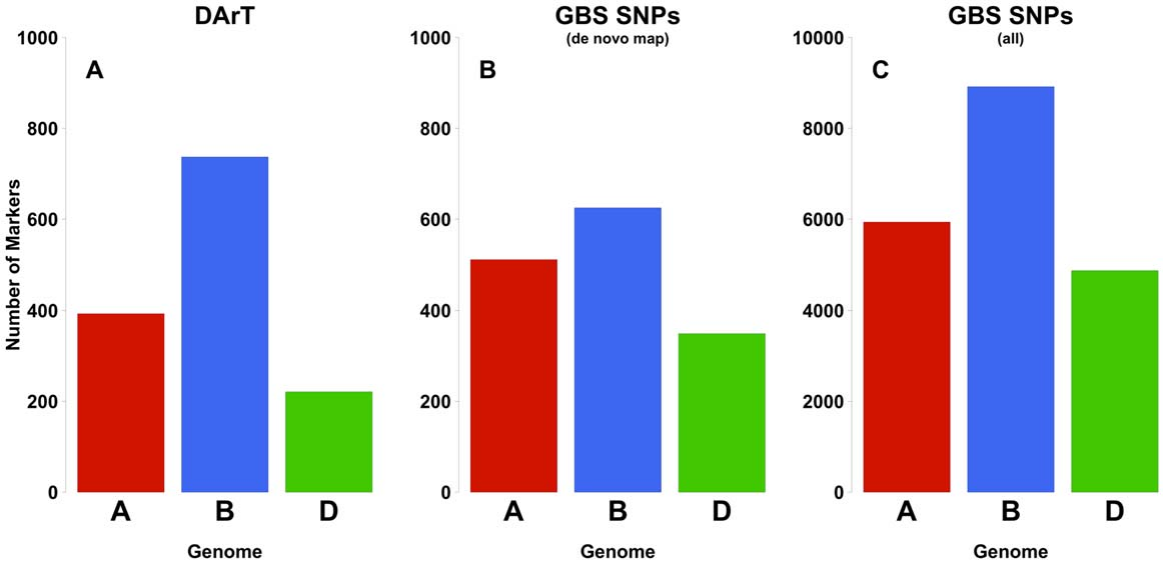
Histogram of number of markers in the three wheat genomes for DArT and GBS SNP genetic maps. A) The number of markers assigned to each genome from the DArT genetic map [25] and B) the number of markers in each genome from the *de novo* genetic map constructed using GBS SNP markers and the AntMap Algorithm. C) The total number of SNPs assigned to each genome using the bin mapping approach in SynOpDH (note different units on vertical axis).

**Figure 4:**
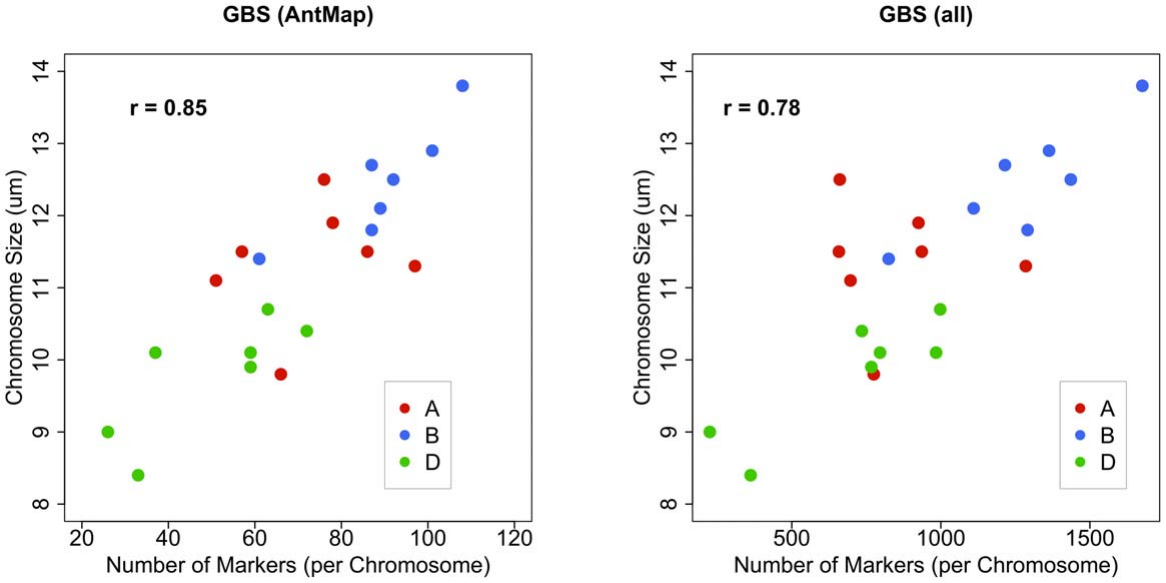
Marker number per chromosome correlates with physical chromosome size. The size of each hexaploid wheat chromosome [28] was compared with the number of genotyping-by-sequencing SNP markers on that respective chromosome. Using AntMap the *de novo* constructed map has 1,485 GBS SNP markers while the full set of mapped GBS markers is 19,720. The correlation coefficient between the marker number and the chromosome size is shown in the upper left of each graph. The legend shows the color-coding for chromosomes from each genome.

In the wheat SynOpDH population marker density on the reference map was initially lower. We therefore first explored development of a genetic linkage map *de novo* using only the GBS SNP markers. Presumably, due to a larger genome and lower sequence coverage of the GBS tags in the wheat population, we only observed 1,771 putative SNPs that had less than 20% missing data. We selected a set of 1,491 SNP markers where there were low levels of missing data and the SNP call was present in the parents and of the opposite allele. We then used AntMap to construct a genetic linkage map of the 21 wheat chromosomes [27]. A subset of DArT markers spaced >20 cM along the chromosome were used to anchor the GBS SNP map onto respective chromosomes. We assembled 1,485 of the SNP markers into a genetic linkage map of 21 linkage groups. There were no inconsistencies between the DArT and the GBS linkage groups. We observed higher numbers of GBS SNP markers on the D-genome compared with that of the DArT map, though the number of D-genome markers was lower than either the A or B-genomes (Figure 3). The marker number of for each chromosome, however, was well correlated to the physical chromosome size (Figure 4) [28]. Using this newly developed GBS SNP map, we then mapped 19,720 SNP markers using the bin mapping approach (Dataset S3) and 367,423 tags as dominant markers.

In the SynOpDH population we observed a deletion on Chr. 2D with the GBS data (Dataset S2). This deletion was present in some lines that inherited the telomeric region of 2D from the synthetic parent. However, this deletion was not present in all of the SynOp DH lines, only a set of those originating from common F_1_ plants. The presence of this deletion among some but not all of the DH progeny indicates heterogeneity for the deletion in the original parental seed stocks of the synthetic parent. We also observed the deletion in an additional DH line that was derived from a different F_1_ than the F_1_ plants for the first set. Based on other DH progeny, this F_1_ plant should have carried the full 2D chromosome, indicating the DH line originated from a different F_1_ than indicated by the pedigree record.

## Discussion

We developed a modified GBS approach using two enzymes and a Y-adapter to generate “uniform” GBS libraries where Adapter 1 and Adapter 2 are on opposite ends of every fragment. To utilize *PstI* as a rare-cutting restriction enzyme we first developed a set of GBS barcodes compatible with the *PstI* restriction site and overhang. Using this algorithm we also designed a set of 384-barcodes for *PstI* that are being used for subsequent experiments (Table S2). As with the original GBS protocol, the approach described here can be used with a ranged of different restriction enzymes to produce a higher or lower complexity reduction of the genome being assayed.

Using this genotyping-by-sequencing approach on bi-parental double haploid populations, we identified and genetically mapped 34,000 SNPs in barley and 20,000 in wheat. These high-density maps will contribute to a fundamental knowledge of the genome structure and have numerous applications in genomic research. High-density maps are valuable in applied breeding programs to enable genomic selection and precise mapping of agronomically important genes for marker-assisted selection. Although we identified tens of thousands of SNP markers in these populations, the resolution of the genetic maps now becomes limited due to relatively small population size. Generation of GBS maps on other bi-parental populations and integration into a common reference will enable the development of a species reference map incorporating hundreds of thousands of GBS SNPs and give a picture of haplotypic diversity and genome structure across populations. The high-density reference maps developed with GBS can then be integrated with whole-genome shotgun sequencing contigs as well as BAC-end sequence to anchor and order the reference genomes. This approach will create a positive feed-back loop: as the reference genome develops (assisted by GBS maps) contextual ordering of reference sequence will enable better SNP calling in the GBS data-sets and haplotypic imputation of any missing data.

The GBS data enabled observations of several genomic features in the barley and wheat populations. Analysis of the OWB GBS data highlighted structural features such as the probable genetic position of the centromere for each chromosome. This was evidenced by a high number of GBS markers falling into one or a few recombination bins. This trend was also evident in the OWB SNP marker map [24] though not as pronounced as observed here in the GBS map. The high number of GBS markers in the recombination bin(s) containing the centromeres indicates that the GBS markers are likely to be somewhat uniformly spaced along the physical chromosome. With reduced recombination around the centromere, it could be expected that this region would encompass a larger physical distance and more GBS markers.

In the OWB population we also observed a region of approximately 18 cM that did not have any informative markers. The lack of informative markers in a region this large likely indicates a region of identity-by-descent (IBD) between the two parents. The OWB parents were created through introgressions of dominant and recessive alleles into related recurrent parents [29] providing opportunity for such IBD regions to arise.

Through development of GBS maps in the SynOpDH population, we were able to observe a heterogeneous deletion presumably present in the synthetic parental stock. This deletion was present in some but not all of the DH progeny and was found within lines derived from a common set of F_1_ plants. Such deletions will cause map distortion on a marker platform such as DArT where markers and dominant and scored as presence or absence. Further cytological investigation of these DH lines and the original parental stocks can confirm the presence and size of the deletion.

The development of GBS methods and high-density genetic maps represents an important advance in the genomics tools available for these cereal crops that currently lack a reference genome sequence. Further, our cost per sample was less than half the cost of other whole-genome genotyping platforms presenting an attractive option for genomic selection applications in breeding programs where cost per sample is critical. Recent increases in the data-output of the next-generation sequencing machines have further reduced per sample cost by generating the same amount of data per sample using a 96-plex library. As sequencing output continues to increase at a rapid pace, GBS clearly becomes more and more attractive. Furthermore, the advantages of GBS such as *de novo* marker discovery and removal of ascertainment bias in new germplasm will make this the genotyping platform of choice in the future.

## Materials and Methods

**Plant Material:** Two community resource double-haploid (DH) populations were used for genetic mapping. In barley, the Oregon Wolfe Barley (OWB) DH population was developed from a cross between morphologically diverse genotypes that are differentiated by a number of dominant morphological markers [24], [30]. We used a set of 82 DH lines from this population along with replicated samples of each parent (OWBdom and OWBrec, the dominant and recessive parents, respectively) to develop two 48-plex libraries. The OWB lines have been genotyped with 2,832 SNP, DArT (Diversity Arrays Technology Pty Ltd) and SSR markers and a combined reference map is available (www.barleyworld.org). In wheat, we used 147 lines from the Synthetic W9784×Opata M85 DH population [25]. These lines have been genotyped with 1,351 DArT markers. DNA from each populations was extracted from seedling tissue using a standard CTAB protocol [31]. DNA was quantified in plates using PicoGreen and DNA concentrations were normalized to 20 ng/ul.

**Adapters:** A set of 48 barcoded adapters with a *PstI* overhang was designed using a custom script in Java (sourceforge.net/projects/tassel/). The barcodes were designed with the following criteria 1) each barcode must be 2 or more bp different from all other barcodes, 2) barcodes can not contain a run of more than 2 of the same nucleotide, 3) barcodes can not contain or recreate (when ligated) the *PstI* or *MspI* restriction site. The full-set of barcodes was designed to optimize the uniformity of each nucleotide at each position. Nucleotide uniformity was accomplished by designing barcodes of different lengths (4 bp to 9 bp) and selecting nucleotides in the barcode that balanced bases in the restriction site [19]. An example of a barcoded adapter pair (barcode sequence shown with capitol bases):

A01_AAGTGA_top

5′ – gatctacactctttccctacacgacgctcttccgatctAAGTGAtgca – 3′

A01_AAGTGA_bot

5′ – TCACTTagatcggaagagcgtcgtgtagggaaagagtgtagatc – 3′

The full list of barcoded adapters for *PstI* is included in Table S1 and the list of OWB and SynOpDH samples with corresponding barcodes are in Dataset S4. The set of 384 barcodes designed for *PstI* is included in Table S2.

The common adapter was designed as a Y-adapter to prevent amplification of the more common *MspI*-*MspI* fragments and adapter-dimers formed by self-ligation of adapter fragments. During the PCR amplification the reverse primer is identical to the Y-tail of the common adapter and can only anneal if the complimentary strand has first been synthesized from the other end of the fragment containing Adapter 1 (Figure 1).

Standard, unmodified oligos were ordered in complimentary pairs and annealed in a high-salt solution to form the double-stranded adapter prior to use. The adapters were annealed by heating to 95 C and then slowly cooling to 30 C at a rate of −1 C/minute in a BioRad DNA engine by programming a single step PCR cycle at 95 C for 1 minute and then decreasing the temperature by 1 C each cycle for 65 cycles. After ligation, the adapters were quantified using Quant-iT™ PicoGreen® (Molecular Probes/Invitrogen Eugene, OR 97402). The adapters were adjusted to a uniform concentration of 0.1 uM.

**Restriction Digest:** Genomic DNA (200 ng) was digested in 20 ul reaction volume of NEB Buffer 4 with 8 U of *HF-PstI* (High-Fidelity) and 8 U of *MspI* (New England BioLabs Inc., Ipswich, MA 01938). The digest was conducted at 37 C for 2 h and then 65 C for 20 min to inactivate the enzymes.

**Ligation:** The ligation reaction was completed in the same tube/plate as the digestion, again using NEB Buffer 4 with the addition of ATP. For wheat and barley, 0.1 pmol of the respective Adapter 1 (0.1 pmol for 200 ng of genomic DNA) and 15 pmol of the common Y-adapter were added to the samples. A master mix of NEB Buffer 4 (1× final), ATP (1 mM final), and 200 U T4 ligase (NEB T4 DNA Ligase #M0202) were added to each sample. The ligation was completed at 22 C for 2 h and the ligase was inactivated prior to pooling the samples by holding at 65 C for 20 min.

**Multiplexing and Amplification:** Ligated samples were pooled and PCR-amplified in a single tube, producing a single library from 48 samples, which was sequenced on a single lane of Illumina GAII or HiSeq2000. The libraries were amplified for 18 cycles consisting of 95 (30 sec), 62 C (30 sec), 68 C (30 sec).

**Sequencing:** Two 48-plex libraries from the OWB population and four 48-plex libraries from the SynOpDH population were constructed for this study. To construct full libraries, the parents and a few of the DH lines were replicated. The barley libraries were each sequenced on one lane of Illumina GAII and one lane of Illumina HiSeq2000. The first two wheat libraries were sequenced on Illumina GAII and the second two libraries were sequenced on Illumina HiSeq2000. All barley and wheat sequences were submitted to the National Center for Biotechnology Information (NCBI) Short Read Archive (study # SRP009867.1).

**Processing of Illumina Raw Data:** From the (unfiltered qseq) Illumina data, sequences were assigned to individual samples using the barcode sequence and trimmed to 64 bp using a custom script in Java (www.maizegenetics.net, sourceforge.net/projects/tassel/). Only sequences that had an exact match to a barcode followed by the expected sequence of 5 nucleotides remaining from a *PstI* cut-site were kept. The full set of reads was then examined for unique tags that were present in more than five different lines. The tags were then collapsed into a matrix of presence/absence for each sample. This matrix was then used for mapping the tags as dominant markers and internal referencing for SNP calling.

**Figure 5:**
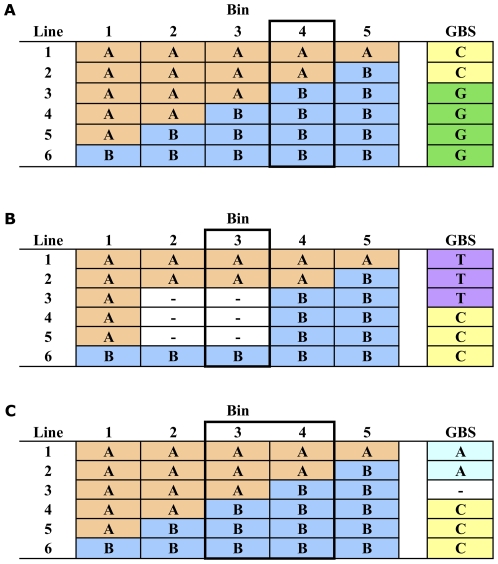
Example of placement of GBS SNP markers into genetic bins of the double haploid mapping populations. A) A GBS SNP without missing data will be precisely placed when all bin genotypes are known (Bin 4). B) When the bin genotypes are “unresolved” (i.e. there is no informative marker in that genetic bin, then a GBS SNP without missing data can again be placed into a single recombination bin in a two step process (Bin 3). C) In the case of missing data in the GBS SNP a range of possible bins (Bin 3–4) were assigned to that GBS SNP.

**Genetic Mapping of SNPs and Tags:** For bin mapping we assumed that marker order in the reference map was correct. The recombination events present within the progeny lines defined the attainable mapping resolution. We define bins such that, for all markers within a bin, the alleles received by a line should have originated from the same parent. This definition also gives the bin's genotype for that line. Thus, if a recombination between a pair of ordered markers occurs in a single line this recombination defines the boundary between two bins. If a recombination between a pair of markers occurs in *two* lines, those two recombination events define both left and right boundaries of a bin. For those two lines, however, the genotype of that bin is unknown. That is, it is unknown whether the recombination present in a line forms the left boundary of the bin (in which case the bin genotype for that line would be that of the right flanking marker) or if it forms the right boundary of the bin (in which case the bin genotype for that line would be that of the left flanking marker). Similarly, if the same recombination occurs in three lines, two bins are defined, and their genotype is unknown in all three lines. For lines with no recombination between the flanking markers, the bin genotype can be inferred because there is no change in the parent of origin in successive bins. A bin for which the genotype of all lines is known we considered “resolved.” Equivalently, one or more markers will be definitively placed in this bin. The algorithm placed a GBS SNP into a bin if the SNP allele matched the bin genotype for all lines where the bin genotype was known. Unknown bin genotypes result in a GBS SNP being consistent with a range of bins. Once an initial range was determined, it was narrowed by counting the number of recombinations from the last resolved bin, as illustrated Figure 5A. When there was no missing data in either the bin map or the GBS SNP data, the GBS marker is located into bin 4 in a single step for a perfect match between the GBS SNP marker and the parent of origin after phasing the GBS SNP.

In the case of missing data in the bin map due to unresolved recombination bins, GBS markers were placed into exact bins in two steps. Referring to Figure 5B, in step 1, the GBS marker would be placed in both bins 2 and 3. In step 2, the GBS marker would be determined to be two recombinations away from bin 1 (lines 4 and 5) and one recombination away from bin 4 (line 3). This step would move the lower bin to 3 (bin 1+2 recs) and the upper bin also to 3 (bin 4−1 rec). For this GBS marker without missing data the exact recombination bin can be determined. In this example bin map, Bin #3 would now be “resolved” as the genotype for all lines is known.

For GBS SNPs with missing data in lines with informative recombinations (Figure 5C) the GBS SNP was placed in a range of possible bins. In the example of 5 C it is unknown if line #3 carries the A or the C allele. Based on this ambiguous allele assignment the GBS SNP would be assigned to both Bins 3 to 4 (i.e. the range of Bins 3–4).

Mapping the SNP markers with low levels of missing data resulted in an improved genetic bin map. This improved bin map was then used to map all GBS tags as presence/absence dominant markers. A binomial test was used to determine the maximum likelihood bin for each tag [19]. Parameters for the binomial distribution are the number of trials, the number of successes, and the probability of success. The number of trials was the number of lines carrying a GBS tag. The number of successes was the number of those lines with bin genotype from the same parent. The null hypothesis probability of success was 0.5. A tag was assigned to the bin for which the probability of the null was lowest with a minimum significance <0.001.

## Supporting Information

Text S1 **Genotyping-by-sequencing protocol for** ***PstI*** **-** ***MspI*** **.**
(PDF)

Table S1 **GBS barcodes sequences optimized for 48-plex with** ***PstI*** **.**
(XLSX)

Table S2 **Design of 384 barcodes optimized for** ***PstI*** **.**
(XLSX)

Dataset S1 **GBS SNPs mapped in the Oregon Wolfe Barley Population.**
(TXT)

Dataset S2 **Genetic linkage map of Synthetic W9784×Opata M85 constructed using GBS SNP markers and AntMap algorithm.**
(XLSX)

Dataset S3 **GBS SNPs mapped in the Synthetic W9784×Opata M85 DH population.**
(TXT)

Dataset S4 **Barcodes and samples used for GBS of Oregon Wolfe Barley DH and Synthetic W9784×Opata M85 DH populations.** Additional files of GBS tags mapped as dominant markers in the OWB and SynOpDH populations are available from the corresponding author on request.
(XLSX)
