# Supplementary material for: Development of High-Density Genetic Maps for Barley and Wheat Using a Novel Two-Enzyme Genotyping-by-Sequencing Approach
Source: PLoS One. 2012 Feb 28;7(2):e32253. doi: 10.1371/journal.pone.0032253 (PMC3289635; doi:10.1371/journal.pone.0032253)
Supplement: Text S1 — Genotyping-by-sequencing protocol for PstI - MspI . (PDF) [file pone.0032253.s001.pdf]

PstI-MspI GBS

Genotyping-by-sequencing Protocol PstI-MspI

J. Poland, USDA-ARS / Kansas State University

Patrick Brown, University of Illinois

20 Dec 2011

ADAPTERS

The adapters are ordered as normal oligos (IDT with standard desalting; 25uM amount). For each adapter two oligos are ordered in complimentary pairs and must be annealed to form the double stranded adapter prior to use of the protocol. They are very stable after annealing and can be stored at -20 indefinitely. The adapters are annealed by heating to 95C and then slowly cooling to 30C at a rate of -1C/minute. This was accomplished in a BioRad DNA engine by programming a single step PCR cycle at 95C for 1 minute and then decreasing the temperature by 1C each cycle for 65 cycles. The barcoded adapters can be ordered in a plate and all of the annealing completed in plate format. The adapters should be quantified after annealing to ensure that the double strand DNA formation was complete and they are at the correct concentration. Uniform concentration of adapters is critical to producing uniform numbers of reads between samples when sequencing the multiplexed library.

QUANTIFY AND NORMALIZE DNA

DNA concentration is critical to producing even numbers of sequence tags from each sample. It is recommended that DNA be quantified using a florescence based quantification method such as PicoGreen.

RESTRICTION DIGEST

This protocol uses a double-digest with *PstI* and a second enzyme *MspI*. The *PstI* overhang corresponds to Adatper1 (barcoded) and the second enzyme overhang corresponds to the common Adapter2 (Y-adapter). The sequencing reaction proceeds from Adapter1 and sequences through the barcode. The generated libraries can be sequenced from both ends though paired-end reads are generally not used.

NEB enzymes are used for the double-digest as they are optimized to work in the same buffer. *PstI* and other common enzymes are used in NEB Buffer4.

LIGATION

The ligation is completed in the same tube/plate as the digestion. The ligation reaction is conducted in NEB Buffer4 with the addition of ATP (NEB T4 DNA Ligase #M0202). A very high concentration of T4 is used to ensure adequate ligation of all the fragments. The concentration of Adapter1 needs to be adjusted depending on the species. For wheat and barley 0.1 pmol is close to the optimal amount for 200ng of genomic DNA. The Adapter2 is a Y-adapter and can be added in excess, as it will

not amplify unless the PCR reaction has first proceeded from Adapter1 on the other end of the same fragment. The ligase should be inactivated prior to multiplexing the samples by holding at 65C for 20min after the ligation is complete.

NOTE: 50-100x more common Adapter2 than Adapter1. Adapter 2 is the Y-adapter so it can be added in excess.

MULTIPLEXING

The ligated samples are multiplex and PCR amplified in a single tube. This produces a single library from 96 samples, which is sequenced on a single lane of Illumina HiSeq.

PCR AMPLIFICATION

The multiplex library is PCR amplified using a short extension time. This will enrich for fragments that are in the 200-500bp range and suitable for bridge amplification. Only fragments that have a *PstI* cut-site and an *MspI* (or second enzyme) cut-site will amplify. The *MspI-MspI* fragments will be common but will not amplify due to the Y-adapter. The *PstI-PstI* fragments will be very rare.

Multiple PCR reactions are performed for each library to help reduce any random over amplification that might occur during a single reaction. The different reactions are then pooled for the final library.

***PstI-MspI* GBS**

Anneal Adapters

1X Elution Buffer (EB) – 10mM Tris-Cl, pH 8.0-8.5
10X Adapter Buffer (AB) – 500mM NaCl, 100mM Tris-Cl

This is completed in plates for the barcoded adapters (Adapter1)

1. Suspend (dried) single-stranded adapter oligos to 100uM in 1X EB
2. Make 100ul of 10uM double stranded adapter:
   10ul – each single stranded oligo (@ 100uM)
   10ul – 10X AB
   70ul – H_20
   Heat to 95C and cool at 1C per/minute to 30C. Hold at 4C.
   (program "anneal")
3. Dilute adapters 3:10 to ~3uM and quantify using PicoGreen (~50ng/ul).
4. Normalize to 1.6 ng/ul (=0.1uM) *Note: These are the ng/ul concentrations for the short version of Adapter 1. See below. Full length adapters will need to be a higher ng/ul concentrations for the same molarity.*

Make MspI common reverse Y-adapter (Adapter2) - single tubes
Common reverse adapters – follow same steps but leave Adapter2 at 10uM for working stock.

Working Adapter Stock
Each well in the working adapter plate will have 0.02uM (unique) barcode Adapter1 and 3uM (common) Adapter2

In 96-well plate add:
20 ul – Barcoded Adapter1 @ 0.1uM
30 ul - MspI Adapter2 @ 10uM
50 ul – 1X AB
Mix well, spin down

Normalize DNA

1. Quantify DNA using PicoGreen. Protocol "PicoGreenDNAQuant.doc"
   (DNA concentration must be between 20ng/ul and 150ng/ul for accurate pippetting. If too high, dilute before normalizing.)
2. Normalize DNA plate to 10ul @ 20ng/ul (200ng total)

PstI-MspI GBS

<u>Restriction (20ul)</u>

10 ul – DNA (20ng/ul = 200ng total)
10ul – restriction master mix

PstI-HF – NEB #R3140 (20,000 units/ml)
MspI – NEB #R0106 (20,000 units/ml)

Restriction MasterMix:

Plate	Sample
220	2.0 ul – 10X NEB Buffer 4
44	0.4ul – PstI-HF (8 units)
44	0.4ul – MspI (8 units)
792	7.2ul – H_20

Normalize 10ul DNA @ 20ng/ul into 96-well plate.
Add 10ul Restriction MasterMix to DNA and mix.

Digest: 37C for 2 hour; 65C for 20 min; hold at 8C (program "reslig")

NOTE: Proceed directly to ligation.

<u>Ligation (40ul)</u>

20 ul - restriction digest
5 ul - Adapters (0.02uM Adapter1 = 0.1pmol, 3uM Adapter2 = 15pmol)
15 ul - Ligation Master Mix

Ligation MasterMix:

Plate	Sample
220	2 ul – NEB Buffer 4
440	4 ul - ATP @ 10mM (final concentration 1 mM)
55	0.5 ul – T4 DNA ligase (200 U)
935	8.5 ul - H_20

To 20ul restriction digest add 5ul Adapters.

Add 15ul Ligation MasterMix and mix.

Incubate at 22C for 2h; 65C for 20 min; 8C forever (program "ligate-kill-hold")

NOTE: Completed ligation can be stored at -20C.

PstI-MspI GBS

Multiplex

Pool 5ul from each sample ligation to a single tube (Add 20ul to empty tube prior to running robot, total volume = 500ul)
QIAgility program: "Pool 96"

Clean-up on Qiagen column (QIAquick PCR Purification Kit)
For each library do two clean-ups.
1. combine 200ul of pooled Ligation DNA and 1000ul of buffer PB in a fresh tube,
2. add 600ul to column, spin down,
3. add second 600ul to column and spin down
Follow manufacturer's directions and re-suspend in 60ul. Combine the two clean-ups from that library.

Amplification (25ul x 8)

Make 8 PCR reactions for each library.

10 ul – DNA (digested library)
5 ul – 5x NEB MasterMix
2 ul – 10uM Forward & Reverse Primers @ 10uM ("Illumina_PE")
8ul – H_20

PCR ("solexa")
95C (30 sec)
{ 95C (30 sec), 62 (20 sec), 68C (30 sec)* } 16 cycles
72C (5 min)
4C (forever)

For each library, pool the 8 PCR reactions and clean-up on Qiagen column (QIAquick PCR Purification Kit). Follow manufacturer's directions and re-suspend in 30ul

* the length of the PCR extension can be shorted or lengthened to produce the optimal distribution of fragments

Check library on 1% gel or BioRad Experion.

PstI-MspI GBS

ADAPTERS

ADAPTER 1

Barcoded adapters ("Adapter1")
This is the adapter with variable length barcode on the end. An example of the AAGTGA barcode:

```
5' CACGACGCTCTTCCGATCTXXXXXTGCA     GNNNNNNN 3'
3' GTGCTGCGAGAAGGCTAGAXXXXX     TGCACNNNNNNN 5'
```

This is the short version of the barcoded (XXXXX) adapters (Tm=57)

_bot XXXXXagatcggaagagcgtcgtg

_top cacgacgctcttccgatctXXXXXtgca

ADAPTER 2

Common adapters ("Adapter2")
These adapters are designed respectively for the second enzyme used in the double digest. They are designed for the Paired-end sequencing. Adaptor2 is a Y-adaptor so the reverse primer (A2Rprimer) can only bind if the forward primer (A1Fprimer) has extended from other end (a PstI site with Adapter1)
Adapter2 has a 5' CG overhang for MspI. (If an enzyme leaves a 3' overhang (i.e. PstI) a different adapter combination must be designed)

Adapt2(PE) for MspI (C|CGG).

```
5' nnnnnnnnC    CGAGATCGGAAGAGCGGGGACTTTAAGC
3' nnnnnnnnGGC    TCTAGCCTTCTCGCCAAGTCGTCCTTACGGCTCTGGCTAG
```

>Adpt2PE_top_cg
cgAGATCGGAAGAGCGGGGACTTTAAGC

>Adpt2PE_bot
GATCGGTCTCGGCATTCCTGCTGAACCGCTCTTCCGATCT

AvaII (G|GWCC)
>Adpt2PE_top_gtc
gtcAGATCGGAAGAGCGGGGACTTTAAGC

>Adpt2PE_top_gac
gacAGATCGGAAGAGCGGGGACTTTAAGC

PRIMERS

The Illumina primers are identical to the oligos on the Illumina flow cells with the additional complimentary sequence for the Adapter1 (Forward) and Adapter2 (Reverse) respectively.

>IlluminaF_PE (Tm=70) 58bp (Tm=57)
AATGATACGGCGACCACCGAGATCTACACTCTTTCCCTACACGACGCTCTTCCGATCT

>IlluminaR_PE (Tm=70) 46bp (Tm=62)
CAAGCAGAAGACGGCATACGAGATCGGTCTCGGCATTCCTGCTGAA

```
Adapter 1 + primer + barcode = 62-68bp
Adapter 2 + primer = 61bp
Min fragement size = ~125bp
```
